# Glomerular Function and Structure in Living Donors: Lessons from Single Nephron Studies

**DOI:** 10.1007/s40472-016-0092-y

**Published:** 2016-02-11

**Authors:** Colin R. Lenihan, Bryan D. Myers, Jane C. Tan

**Affiliations:** Division of Nephrology, Department of Medicine, Stanford University School of Medicine, Stanford, CA USA

**Keywords:** Living donor, Donor safety, Single nephron, Glomerular physiology, Kidney donor

## Abstract

One third of the kidney transplants performed in the USA come from living kidney donors. The long-term outcome of healthy individuals who donate kidneys is mostly excellent, although recent studies have suggested that living donation is associated with a small absolute increase in the risk of end stage renal failure. Much of our understanding about the progression of kidney disease comes from experimental models of nephron loss. For this reason, living kidney donation has long been of great interest to renal physiologists. This review will summarize the determinants of glomerular filtration and the physiology that underlies post-donation hyperfiltration. We describe the ‘remnant kidney’ model of kidney disease and the reasons why such progressive kidney disease very rarely ensues in healthy humans following uninephrectomy. We also review some of the methods used to determine glomerular number and size and outline their associations.

## Introduction

The first living kidney donation was performed in 1954 [1]. Currently, more than 5000 live kidney donations are performed annually in the USA alone [2]. Since the inception of the practice, our knowledge of the pathophysiology of chronic kidney disease (CKD) and its progression has greatly advanced, based in no small part on animal experimental studies of nephron loss. In this review, we highlight renal physiological and pathophysiological studies that are of special relevance to living kidney donation.

### The Physiology of Glomerular Filtration

Much of our understanding about the dynamics of glomerular filtration stem from experiments performed in the Munich-Wistar rat. The kidneys of this rat strain are endowed with superficial surface glomeruli, permitting in vivo glomerular micro-puncture with direct measurement of single nephron glomerular filtration rate (SNGFR) and glomerular capillary hydraulic pressure (*P*_GC_) [3]. Further insights into glomerular physiology have been gained from studies of humans and large mammals in which SNGFR and *P*_GC_ were either measured indirectly or estimated [4–6].

**Fig. 1:**
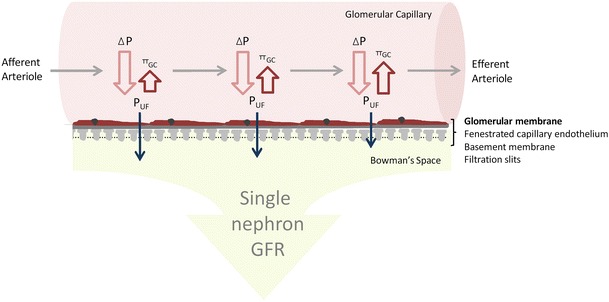
A schematic view of glomerular ultrafiltration across the glomerular capillary. The Starling’s forces at work are the (1) glomerular transcapillary hydraulic pressure *ΔP* which is the difference between the glomerular and Bowman’s Space hydrostatic pressures and (2) the opposing glomerular capillary oncotic pressure (*π*_GC_). Bowman’s Space oncotic pressure is negligible and usually disregarded. The difference between ΔP and *π*_GC_ at any given point is the net ultrafiltration pressure (*P*_UF_). *ΔP* remains constant across the length of the glomerular capillary. However, because *π*_GC_ rises as plasma proteins concentrate, the rate of ultrafiltration decreases along the length of the capillary

Glomerular filtration is dependent on four factors (Fig. 1). (1) The glomerular transcapillary hydraulic pressure (*ΔP*) is the difference between the *P*_GC_ and the hydraulic pressure in Bowman’s space. *P*_GC_ is maintained constant by the relative tones of the afferent and efferent arterioles across a range of physiological blood pressures. The *ΔP* favors the flow of filtrate across the glomerular capillary. (2) The glomerular oncotic pressure (*π*_GC_) is determined by the plasma protein concentration in the glomerular capillary and opposes the formation of filtrate. The *π*_GC_ increases along the length of glomerular capillary as unfiltered plasma proteins become concentrated in a progressively contracted volume of plasma. At any point along the glomerular capillary the difference between *ΔP* and *π*_GC_ is the net ultrafiltration pressure (*P*_UF_). In humans, filtration is thought to occur along the full length of the glomerular capillary, a state referred to as filtration dysequilibrium. (3) Increases in renal plasma flow (RPF) augment glomerular filtration rate (GFR) by blunting the plasma contraction-related rise in oncotic pressure that occurs along the length of the filtering glomerular capillary. (4) The ultrafiltration coefficient (*K*_f_) is determined by the surface area of filtering capillary (*S*) and the hydraulic permeability (*k*) of the glomerular filtration barrier (*K*_f_ = *S* × k). In simple terms, the *K*_f_ describes the flow of filtrate (SNGFR) that will occur at a given ultrafiltration pressure (*P*_UF_), as described by the equation; SNGFR = *K*_f_ × (*P*_UF_). The whole kidney *K*_f_ is the sum of the all the individual glomerular *K*_f_ values. The whole kidney *K*_f_ may be affected by changes in either glomerular number or volume. A range of ‘normal’ values for GFR, RPF, *π*_GC,_ and filtration fraction based on studies of living donors (pre- and post-donation) and healthy volunteers are shown in Table 1.

**Table 1:** Reference values for glomerular filtration rate and its determinants

Measure	Pre-donation (mean±SD)	Post-donation (mean±SD)^d^
Iothalmate GFR per 1.73 m^2^ (*n* = 57)^a^	101±19	66±12
Inulin GFR per 1.73 m2 (*n* = 180)^b^	100±18	–
Renal plasma flow (PAH) per 1.73 m^2^ (*n* = 237)^c^	527±132	322±160
π_AA_ mmHg (*n* = 237)	25.3±2.6	–
Filtration Fraction (*n* = 237)	0.2±0.04	0.21±0.03

^a^From reference [7], pre-operative living kidney donors, age range 23–68 years

^b^From reference [8], healthy volunteers, age range 18–88 years

^c^From references [7] and [8], pre-operative living kidney donors and healthy volunteers, age range 18–88 years

^d^For 26 subjects aged 57±7 years, who were 7.4±2.9 years post-donation

πAA, afferent arteriolar oncotic pressure

### Loss of Nephron Mass

**Fig. 2:**
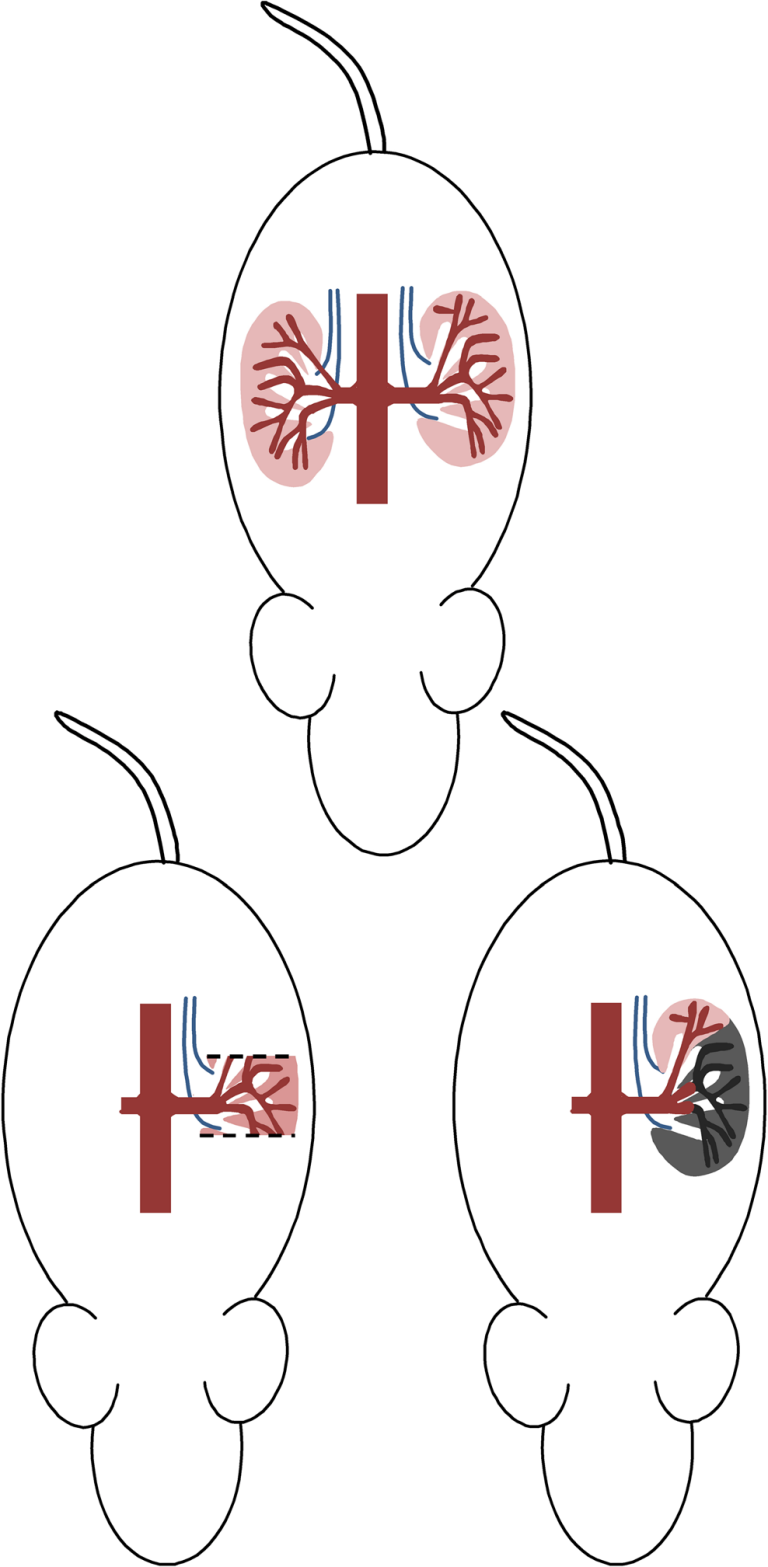
The ‘remnant kidney model’. A 5/6 nephrectomy is achieved through unilateral nephrectomy plus either (1) surgical amputation of the superior and inferior poles of the remaining kidney or (2) ligation of two out of three branches of the renal artery resulting in infarction of two-thirds of the remaining kidney

Experiments performed using the Munich-Wistar rat have also shed light on the pathophysiology of CKD progression. In these studies, a ‘5/6 nephrectomy’ was achieved though uninephrectomy combined with either subtotal infarction or surgical excision of two thirds (or more) of the contralateral kidney (Fig. 2). Subsequent glomerular micropuncture and histological analysis of the remaining kidney revealed compensatory elevation of SNGFR and increases in P_GC_ (glomerular hypertension), single nephron plasma flow and glomerular tuft volume. These rats subsequently developed progressive CKD, the so-called ‘remnant kidney syndrome’, which is characterized by systemic hypertension, proteinuria, and the histological features of focal and segmental glomerulosclerosis (FSGS) [9, 10•]. In humans, a similar phenomenon known as secondary FSGS commonly occurs in kidneys that have already been injured by another process, such as reflux nephropathy [11] or, less commonly, in the setting of obesity [12].

**Fig. 3:**
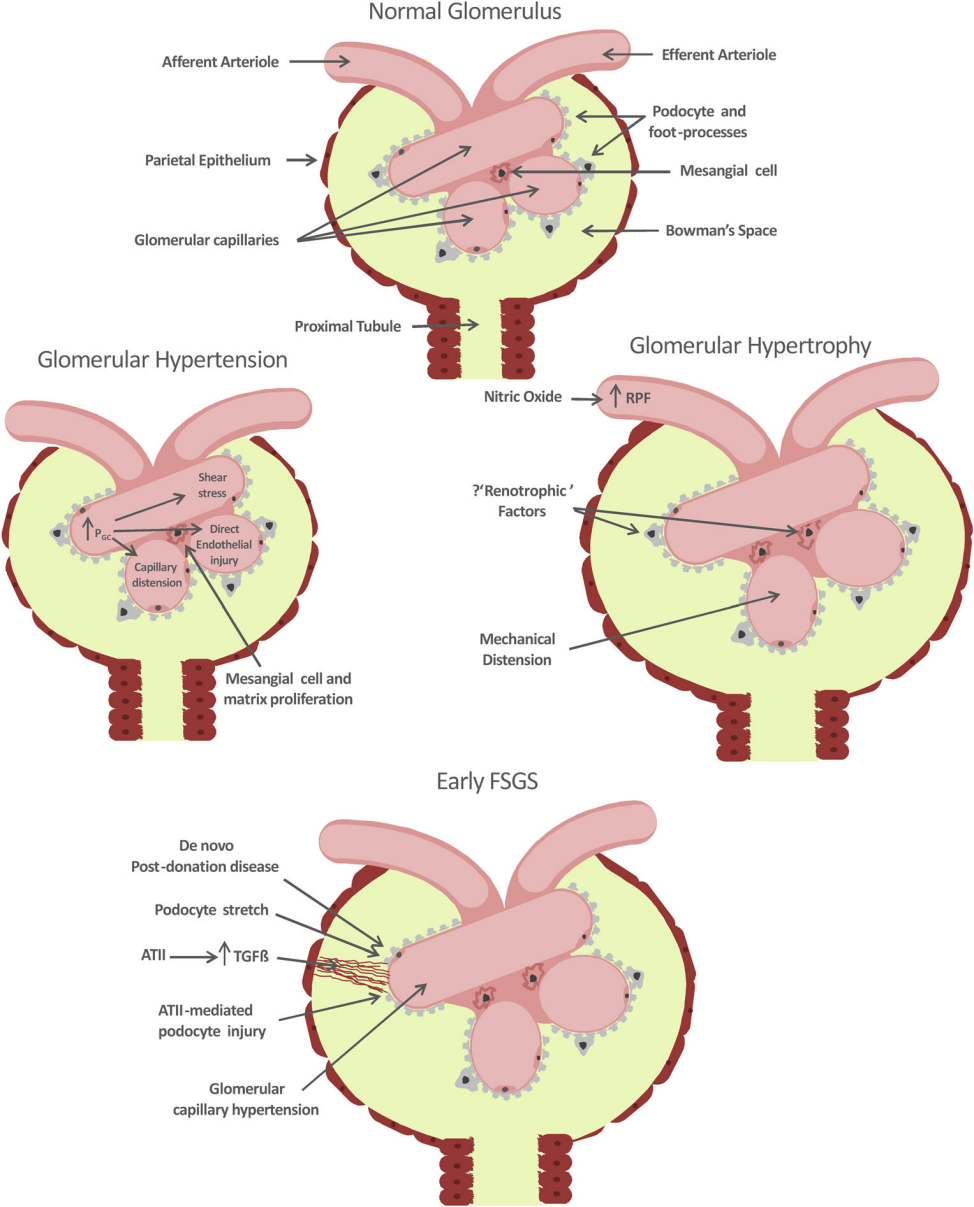
Potential pathways toward the development of focal segmental glomerulosclerosis (FSGS) following loss of nephron mass. *P*_*GC*_ glomerular capillary hydrostatic pressure, *AT*II angiotensin II, *TGFβ* transforming growth factor beta

The pathophysiology of FSGS following the loss of nephron mass has been extensively studied. It has, however, proven difficult to fully disentangle the relative contributions of glomerular hypertension, increased single nephron GFR, compensatory glomerular hypertrophy, and other pathophysiological responses to the development of glomerulopenia-induced glomerular injury (Fig. 3).

Glomerular hypertension alone is almost certainly directly injurious. Experimental studies in the remnant kidney model have shown that normalization of both glomerular and systemic hypertension with an angiotensin converting enzyme (ACE) inhibitor or angiotensin receptor blocker, compared to normalization of systemic hypertension alone using alternative antihypertensive agents, is associated with attenuation of proteinuria and FSGS [13, 14]. Institution of a low protein diet also results in normalization of glomerular pressure and is associated with a similar protection against renal injury [15]. Furthermore, mice chronically treated with an angiotensin II (ATII) infusion develop glomerular hypertension, proteinuria, and FSGS [16]. While these experiments do show an association between raised glomerular pressure and renal injury they do not prove glomerular injury is directly caused by glomerular hypertension. That being said, there are a number of biologically plausible mechanisms for glomerular hypertension-induced renal injury including (1) direct endothelial injury (barotrauma), (2) stimulation of the mesangial cell and matrix proliferation [17], (3) altered glomerular handling of macromolecules leading to their aberrant mesangial deposition, (4) increased podocyte shear stress resulting from increased flow of glomerular filtrate and, (5) mechanical distension of the glomerular capillary necessitating increased podocyte surface area coverage.

An alternative, and not necessarily mutually exclusive, mechanism for glomerular injury following the loss of renal mass centers on the effect of glomerular hypertrophy on podocyte health [16, 18–20]. Podocytes (which are terminally differentiated cells) must enlarge to cover an expanding basement membrane as glomeruli hypertrophy. This process predisposes to the development of gaps between podocytes and the direct exposure of the glomerular basement membrane (GBM) to Bowman’s Space, a process that may initiate sclerosis. Subsequent glomerular loss due to FSGS may in turn result in further hypertrophy of remaining nephrons, setting in motion a vicious cycle of glomerular hypertrophy and further podocyte ‘stretch’. In some experimental models, glomerular hypertrophy alone appears sufficient to result in podocyte death and FSGS [21]. However, glomerular hypertrophy is especially relevant when accompanied by other pathological processes that initiate podocyte injury and loss [19]. Indeed, the combination of glomerular hypertrophy (following uninephrectomy) and ATII (infusion)-mediated systemic and glomerular hypertension results in a significantly greater degree of proteinuria and glomerular sclerosis, than an ATII infusion alone [16]. The link between glomerular hypertrophy and accelerated kidney disease is also supported by a biopsy study showing an association between mean glomerular tuft area and subsequent development of FSGS in patients with minimal change disease [22].

Glomerular hypertrophy occurs rapidly after nephron loss in experimental animal studies [23]. However, the mechanism for ‘compensatory’ glomerular enlargement is not fully understood. Glomerular volume is likely influenced by metabolic workload. For instance, experimental manipulation of diet leads to significant alterations in glomerular volume; both high protein and high salt diets result in increased glomerular volume, while post-nephrectomy glomerular hypertrophy can be prevented by protein restriction [19, 24, 25]. The influence of metabolism and workload on glomerular volume is further evidenced by the strong correlation between body surface area and glomerular volume in humans [26•]. A number of putative ‘renotrophic factors’ have been proposed, including insulin-like growth factor, epidermal growth factor, hepatocyte growth factor, and ATII [27, 28]. ATII does seem to be required for normal glomerular growth during development [28]. However, chronic ATII infusion does not result in glomerular hypertrophy, and the glomerular hypertrophy induced by a high-protein diet is not prevented by ACE inhibition [16, 24]. Direct mechanical stretch resulting from glomerular hypertension may also induce capillary distension and mesangial cell and matrix proliferation [17]. Regardless of its underlying mechanism, nitric oxide-mediated vasodilation, and hyperperfusion of the remaining nephrons appears to be required for ‘normal’ renal hypertrophy to occur following nephrectomy [29].

ACE inhibitors and ATII receptor blockers exert a protective effect in the remnant kidney model [9, 14]. There has been much work examining potential non-hemodynamic role(s) for ATII in the pathogenesis of FSGS. Angiotensin II in certain circumstances may directly injure podocytes. Overexpression of ATII receptors in podocytes is associated with their dysfunction and loss [20]. Moreover, in 5/6 nephrectomized rats, ATII blockade is associated with reduced podocyte injury and loss [30]. In addition, ATII exposure results in increased mesangial cell production of the pro-fibrotic cytokine TGFβ in vitro, while ATII administration is associated with enhanced glomerular TGFβ expression in otherwise healthy rats in vivo [31]. Furthermore, enhanced TGFβ expression occurs in concert with ATII expression in rat glomeruli following 5/6 nephrectomy and ATII receptor blockade significantly reduces post-5/6 nephrectomy glomerular TGFβ expression [32, 33]. The process of secondary FSGS in the remnant kidney is also accompanied by inflammation and it is likely, as with many pathophysiological processes, that a dysregulated immune response may exacerbate tissue injury [34].

### Post-Donation Glomerular Physiology

In humans there are rapid parallel increases in RPF and single kidney GFR following kidney donation, such that GFR falls to 70 % rather than 50 % of its pre-donation level [35]. The early increase in RPF results, at least in part, from nitric oxide-mediated vasodilation [29]. However, an increase in kidney volume of around 30 % [36, 37] also occurs post-donation, which presumably reflects underlying glomerular hypertrophy. Our recent study reported on serial physiological and radiological measurements in 21 living kidney donors followed to a median of 6.3 years post-donation. Mathematically modeled glomerular ultrafiltration dynamics were studied at pre-, early-, and late-post-donation time points. The final model, which assumed that post-donation whole kidney *K*_f_ increased in proportion to renocortical volume, suggested that post-donation hyperfiltration could be entirely attributed to enhanced glomerular ultrafiltration capacity, without any measurable contribution by glomerular capillary hypertension [37].

### Nephron Number and Glomerular Volume

Nephron number in adult humans is highly variable but has been estimated to average 600,000 per kidney [26•]. Nephron number has a positive correlation with birth weight [38], and congenitally low nephron endowment has been proposed as a risk factor for the development of hypertension and renal disease, the so called “Brenner hypothesis” [39].

GFR decreases with age, at an average rate of −0.75 mls/min/1.73 m^2^/year [40]. In parallel, age-related glomerulosclerosis, traditionally attributed to intrarenal vascular disease, increases with age [41, 42]. Autopsy studies of nephron number, such as that of Nyengaard et al., that have counted glomeruli irrespective of whether they are open or sclerosed and have shown a decline in total glomerular number with age suggesting that a proportion of glomeruli are ultimately reabsorbed and effectively disappear [26•]. The inverse correlation between age and glomerular number has also been demonstrated in living donors [7]. The relation between glomerular volume and age is much less clear. However, studies that have excluded sclerosed or involuting glomeruli show a positive correlation between glomerular volume and age [43]. Hypertension may contribute to nephron loss or may be a product of congenital low nephron number. Some studies have suggested that hypertension is associated with low nephron number and large glomerular volume, with differing conclusions, likely, in part, reflecting differences in the populations studied [44–46].

The gold standard for the estimation of glomerular number is the fractionator-dissector method in which a random unbiased (but known) fraction of kidney sections is selected for counting (for a detailed description of the methods see references [26•, 47]). Within each sampled section a counting grid is superimposed and glomeruli within that area (a known fraction of the kidney) are counted. Each counting grid contains two adjacent ‘inclusion’ and ‘exclusion’ lines such that objects touching the inclusion and exclusion lines are counted and not counted, respectively. For each sampled section the succeeding ‘look-up’ section is selected and the same counting frame is identified within that section. Only glomeruli identified in the sampled section but not the look-up section are counted. The relative fractions of cortical area and glomerular area in each section are calculated using point counting. The volume fraction of glomeruli (Vvglom/cor) is the proportion of the sampled area that contains glomeruli divided by the proportion that contains cortex. The numerical density of glomeruli in the cortex (Nvglom/cor) is calculated as the total number of glomeruli counted divided by the total cortical volume of the sampled sections (calculated as the proportion of the sampled area that contains cortex × sampled area × section thickness). The mean glomerular volume is then derived by dividing the Vvglom by the Nvglom. The major disadvantage of this method is that it requires a whole kidney, is labor intensive, and requires consistent and expert tissue processing capabilities. In addition, the calculation of mean glomerular volume will not take into account variation in glomerular volume within a kidney.

Glomerular volume can also be measured directly. The gold standard for direct volume measurement uses the Cavalieri principle, which requires no assumptions about the shape of the glomerulus but requires measurement of multiple serial sections of the same glomerulus [48]. Other methods such as that of Weibel and Gomez, assume the glomerulus is spherical, allowing the mean glomerular area to be calculated from multiple glomeruli (usually > 20) measured through random planar sections [49]. Alternatively, the identification of and measurement of the maximum planar area of a glomerulus through examination of serial sections is significantly less work than the Cavalieri method but also requires the assumption of a spherical glomerulus [48].

**Fig. 4:**
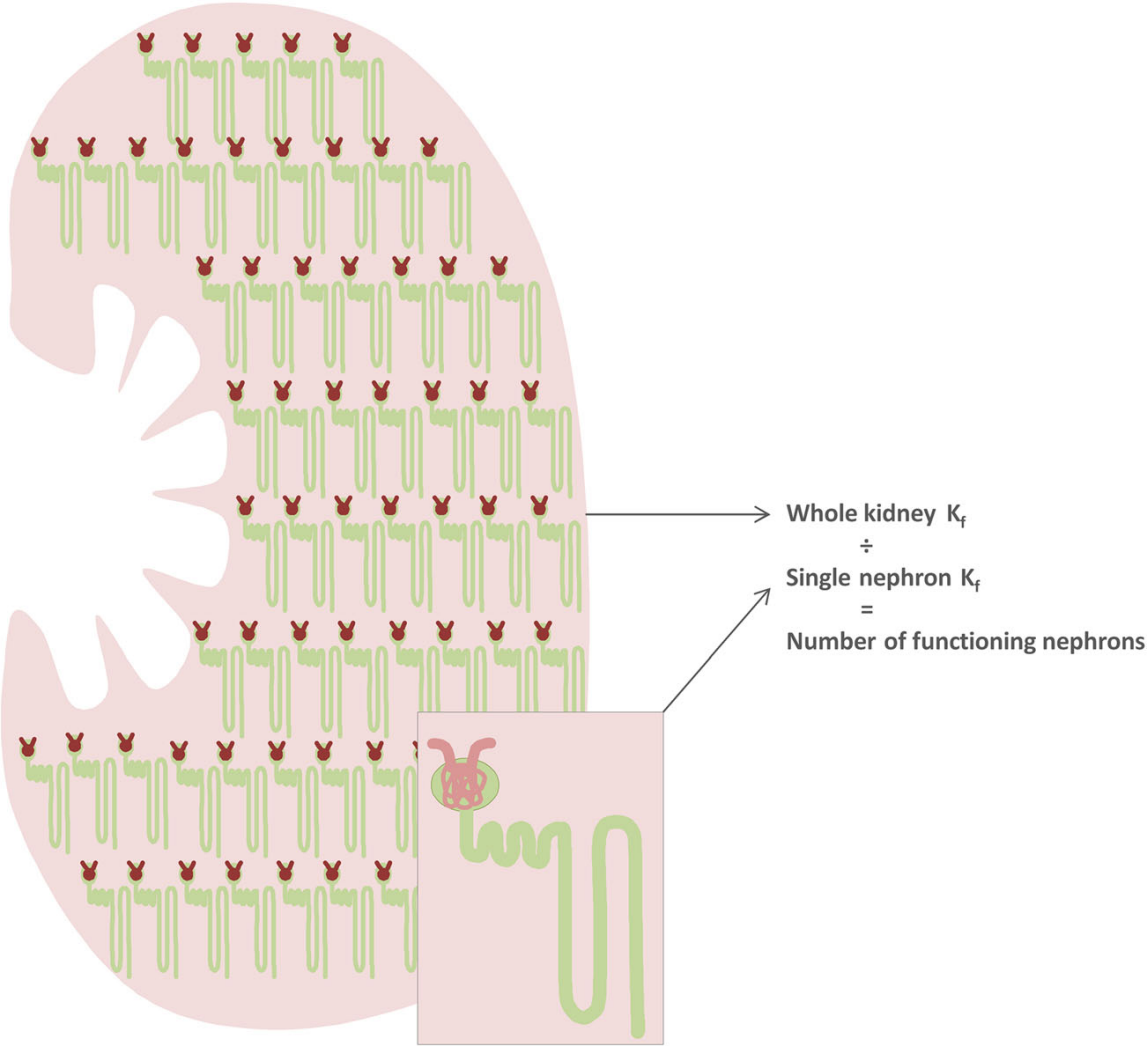
Estimation of the number of functioning nephrons. Whole kidney *K*_f_ is calculated from measured GFR, RPF, and plasma oncotic pressure. Single nephron *K*_f_ is estimated from the histologically and ultrastructurally-derived estimations of filtering surface area and hydraulic permeability in glomeruli obtained through kidney biopsy. The number of functioning glomeruli is then computed by dividing whole kidney *K*_f_ by single kidney *K*_f_

Our group has described a method for estimating the number of functioning glomeruli using kidney biopsy data combined with measurement of GFR, RPF, and plasma oncotic pressure (Fig. 4) [7]. SNK_f_ is a product of glomerular capillary filtering surface area and hydraulic permeability. Filtering surface area is estimated on biopsy specimens by measuring both the volume and percentage filtering capillary surface area of sampled glomeruli. The hydraulic permeability is estimated from the measured GBM thickness. Whole kidney *K*_f_ is estimated using a formula derived by Deen et al., using GFR, RPF, plasma oncotic pressure, and an estimate of *ΔP* [50]. The number of functioning glomeruli may then be calculated by dividing the whole kidney *K*_f_ by the single nephron *K*_f_. The advantages of this method are that it requires only a biopsy sample rather than one whole kidney. The major disadvantage is that the whole kidney *K*_f_ may be influenced by transient hemodynamic changes.

Overall, living donor outcomes are excellent. Most studies have found no increase in all-cause or cardiovascular mortality among donors compared either with the general population [51] or with selected healthy non-donor controls [52–54]. However, two recent retrospective studies have suggested, that when compared to healthy controls screened to simulate donor selection, kidney donation is associated with a small absolute increase in the risk of end stage renal disease (ESRD) [55, 56•]. Studies of post-donation hypertension have disclosed mixed results [57, 58]. However, a meta-analysis has shown that after a median follow-up of 5 years, living kidney donation was associated with a 5-mmHg increase in mean arterial pressure [59].

Historically, concern regarding the outcome of living donation was driven by the huge body of animal experimental work highlighting the adverse effects of nephron loss [60]. However, it is now more than 60 years since the first living kidney donation and while there have been isolated reports of secondary FSGS ensuing late post-kidney donation [60], no epidemic of a post-donation remnant kidney-type syndrome has been identified. There are a number of potential explanations for this; 1) the experimental models of nephrectomy likely overestimate the risk of secondary FSGS. Most of the studies were performed in the rats with quite profound nephron loss resulting from 5/6th (or even 11/12^th^[10]) nephrectomy rather than uninephrectomy. The 5/6th nephrectomy is frequently achieved by combining uninephrectomy with ligation of two out of three branches of the renal artery resulting in subtotal infarction of the remaining kidney, a procedure that is associated with accentuation of the disease process in the remnant kidney when compared to surgical two-third nephrectomy of the remaining kidney [61]. Moreover, the rat appears to be unusually susceptible to the development of secondary FSGS. FSGS is commonly observed following uninephrectomy in rats and is also observed in otherwise healthy aging rats [62–64]. (2) Given the relatively short period of follow-up of humans, it is conceivable that the remnant kidney phenomenon could ensue in a greater proportion of living donors with time. This concern is especially relevant in very young donors, who may reasonably expect to enjoy another 60 years of post-donation life. (3) We infer that, in the absence of a ‘second hit’, post-donation nephron number is well above the threshold required for the remnant kidney phenomenon to occur. However, certain groups such as older donors or hypertensive donors, whose pre-donation nephron number may be at the lower end of the normal spectrum, might be expected over time to have a higher risk of post-donation problems. Indeed, the effect of relative post-donation ‘glomerulopenia’ may contribute to the very small excess risk of ESRD in older compared to younger donors [56•]. Finally, whereas hypertensive donors have shown good renal function and blood pressure control in the short term [65, 66], a lack of longer term data requires that extended follow up of hypertensive donors be undertaken to ensure their long term well-being.

Kidney size correlates with nephron number and has been used as a surrogate marker of nephron endowment [26•]. Smaller kidney size, especially if mismatched with recipient size, has consistently been shown to be associated with poorer graft function and survival in kidney transplant recipients, who in contrast to living donors, are exposed to a myriad of potential (ischemic, immune, toxic) ‘second hits’ [67–69].

Another concern stemming from experimental studies is that larger pre-donation glomerular volume followed by post-donation glomerular hypertrophy could predispose to podocyte injury and the development of post-donation FSGS. To our knowledge, there are no data to date linking pre-donation glomerular volume with kidney donor outcomes. However, in stable kidney transplant recipients, larger glomerular volume measured in early post-transplant protocol biopsies is associated with reduced graft survival [70].

One of the most important advances in nephrology over the past decade has been the identification of the APOL1 genetic risk variants and their association with the development of non-diabetic kidney disease (including FSGS) in patients of African ancestry [71]. The mechanism behind APOL1-associated renal disease has not been elucidated, although the relation between APOL1 risk variants and podocyte injury is being investigated [72]. In the absence of kidney disease, there does not appear to an obvious renal phenotype associated with the APOL1 risk variant. In a recent autopsy study neither glomerular number or volume differed according to APOL1 risk profile, although a potential association between age-related reduction in glomerular number and the APOL1 risk variant was proposed [73]. Kidney transplant survival is significantly reduced in patients who receive a deceased donor kidney that is homozygous for the APOL1 risk variant [74]. The outcome of living donors who are homozygous for the APOL1 variant is unknown; however, it is tempting to speculate that much, if not all, of the excess risk of renal failure in African American donors may be attributable to APOL1 risk variant [56•].

## Conclusion

Historical concerns regarding the safety of living kidney donation are threefold. The first concern was that the metabolic effect of a 30 % reduction in kidney function would prove harmful in the long-term. To date, a significant ‘uremic’ effect associated with kidney donation has not been identified, although, compensatory changes in the phosphate-PTH-FGF23 axis have been cited as evidence of CKD [58]. Secondly, the association between nephron loss and FSGS in experimental models raised obvious concerns about the safety of nephrectomy for kidney donation in otherwise healthy humans. Fortunately, 60 years of living kidney donation has largely allayed fears for an epidemic of post-donation FSGS. The final concern was (and remains) that prior uninephrectomy may accelerate the progression of de novo post-donation kidney disease. This notion is supported by animal models where a reduction in nephron number accentuates most disease processes in the remaining kidney [16, 18, 19]. We believe that a careful pre-donation medical assessment remains vital and hope that new diagnostic developments, such as apolipoprotein L1 G1 and G2 risk variant testing, and improved baseline risk prediction [75] will further enhance our ability to identify and exclude donor candidates who are at high risk of future renal disease.
